# Supplementary figures and images for: Structural basis of Toxoplasma gondii perforin-like protein 1 membrane interaction and activity during egress
Source: PLoS Pathog. 2018 Dec 4;14(12):e1007476. doi: 10.1371/journal.ppat.1007476 (PMC6294395; doi:10.1371/journal.ppat.1007476)

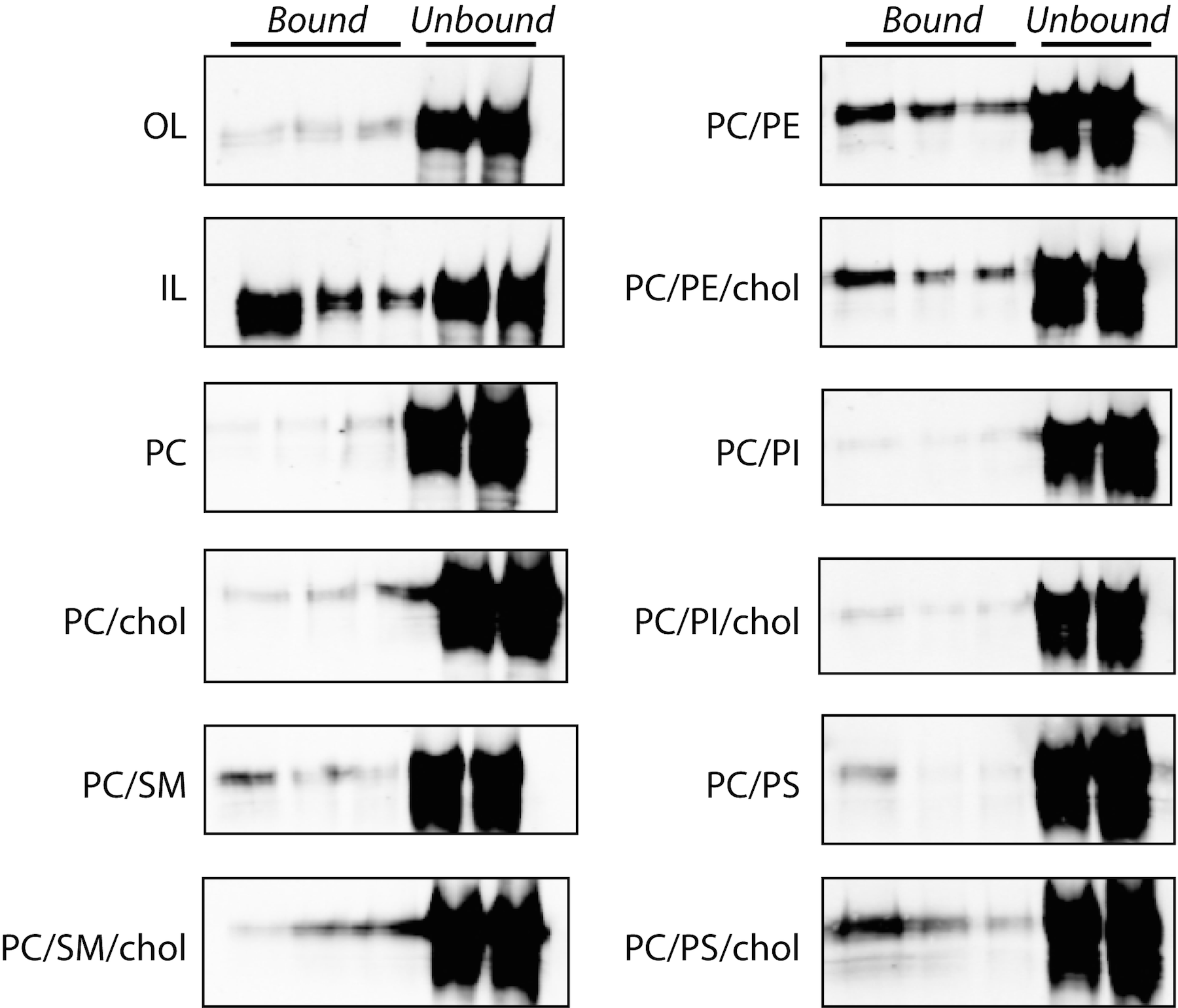

Supplement: S1 Fig — Representative western blots of liposome membrane flotation assays. Liposome composition as follows: OL: 75% PC, 8.3% SM, 16.7% cholesterol; IL: 35.7% PE, 14.3% PS, 21.4% PI, 28.6% cholesterol; PC: 100% PC; PC/chol: 50% PC, 50% cholesterol; PC/SM: 50% PC, 50% SM; PC/SM/chol: 30% PC, 50% SM, 20% cholesterol; PC/PE: 50% PC, 50% PE; PC/PE/chol: 30% PC, 50% PE, 20% cholesterol; PC/PS: 50% PC, 50% PS; PC/PS/chol: 30% PC, 50% PS, 20% cholesterol; PC/PI: 50% PC, 50% PI; PC/PI/chol: 30% PC, 50% PI, 20% cholesterol. Quantification of these bands is shown in Fig 1D. (TIF) [file ppat.1007476.s001.tif]

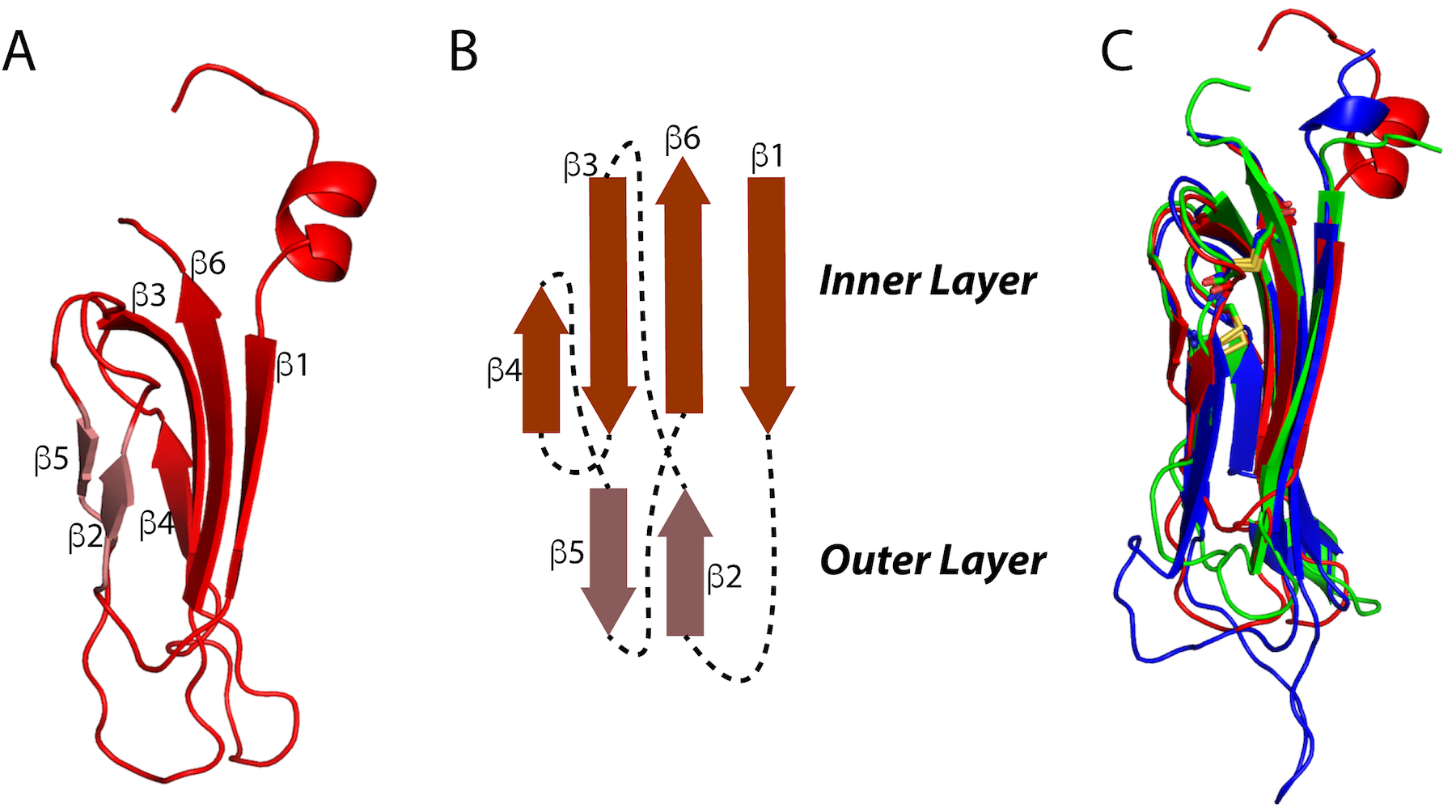

Supplement: S2 Fig — A. Subdomain 1 of the TgPLP1APCβ crystal structure. Each subdomain is made up of an inner β-sheet of four anti-parallel strands and an outer β-sheet of two anti-parallel strands. B. Schematic representation of the inner and outer β-sheets. C. Overlay of the three subdomains of the APCβ crystal structure. (TIF) [file ppat.1007476.s002.tif]

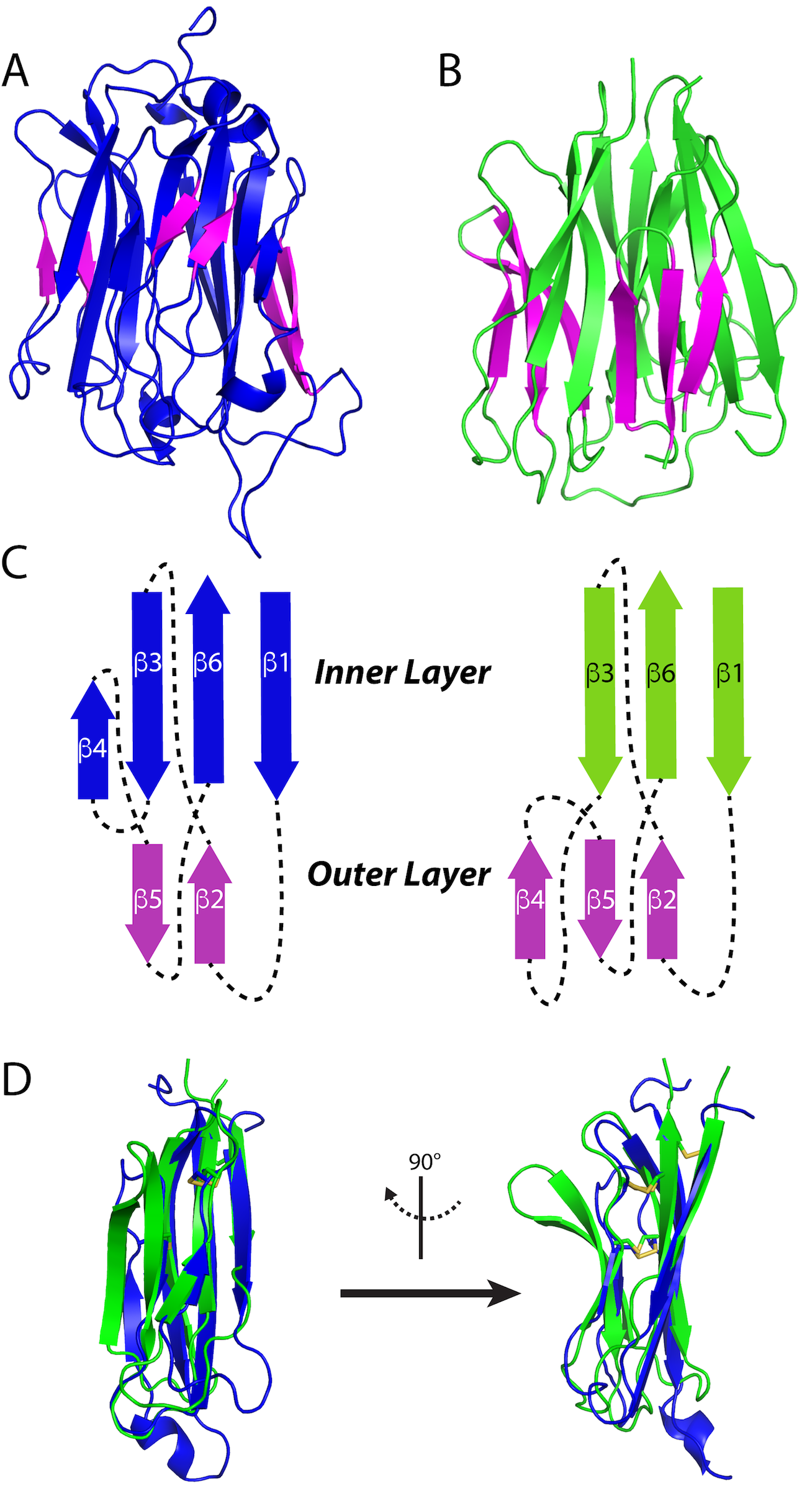

Supplement: S3 Fig — Comparison of the TgPLP1 APCβ crystal structure and the PCSK9 V-domain crystal structure A. Cartoon representation of TgPLP1 APCβ structure. The outer layers of the double layer β-prism are highlighted in magenta. B. Cartoon representation of the PCSK9 V-domain. Similar to panel A, the outer layers of the double layer β-prism are highlighted in magenta. C. Schematic representation of the inner and outer β-sheets in TgPLP1APCβ (left) and PCSK9 V-domain (right) subdomains. D. Overlay of a single subdomain from TgPLP1APCβ (blue) and PCSK9 V-domain (green). Disulfide bonds are shown in stick representation (yellow). (TIF) [file ppat.1007476.s003.tif]

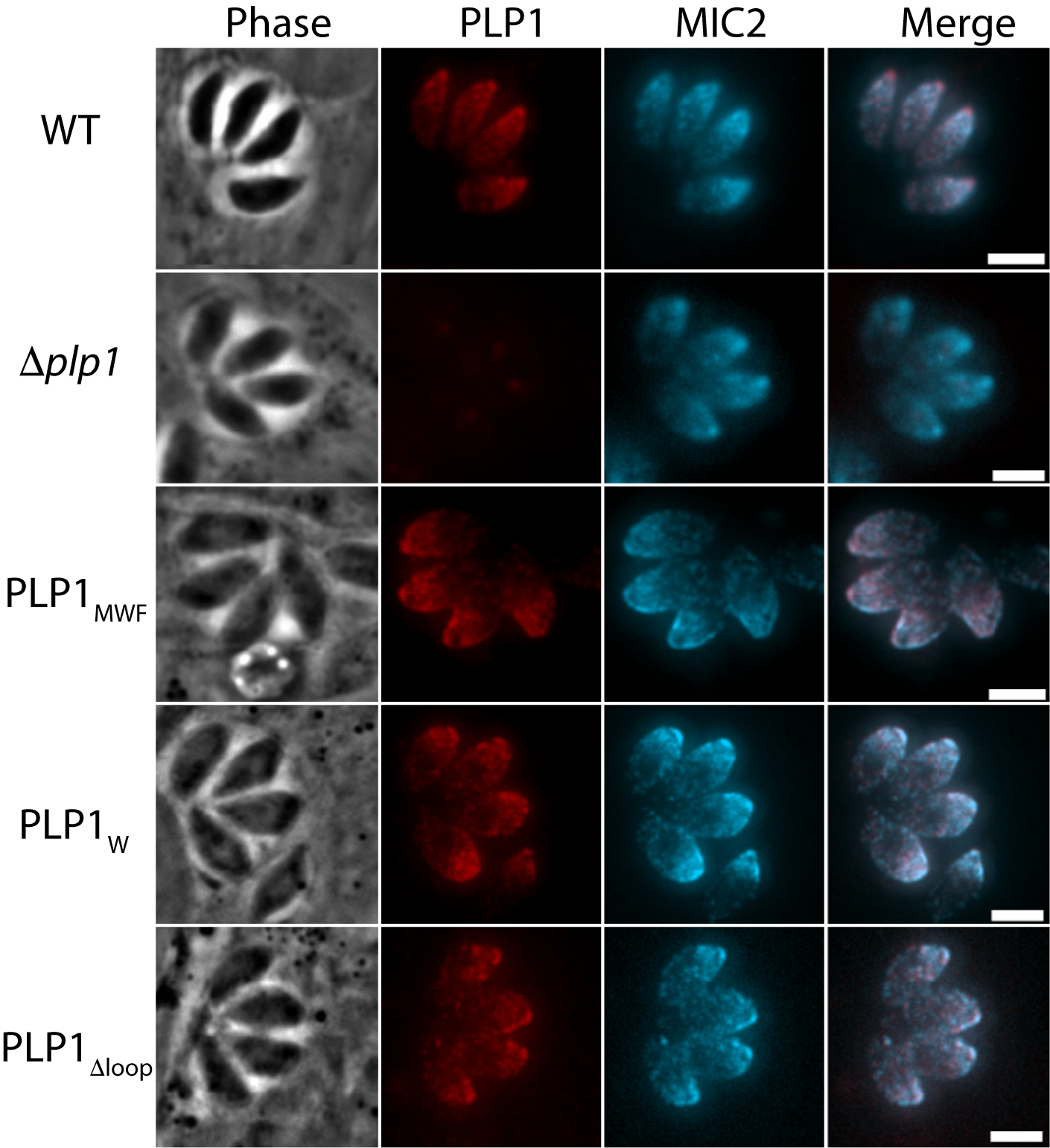

Supplement: S4 Fig — Host cells were inoculated with mechanically lysed parasites and incubated for 30 hours at 37°C. Immunofluorescence microscopy was performed by staining with mouse anti-TgMIC2 (cyan) and rabbit anti-TgPLP1 (red) antibodies and imaged via immunofluorescence microscopy. (TIF) [file ppat.1007476.s004.tif]

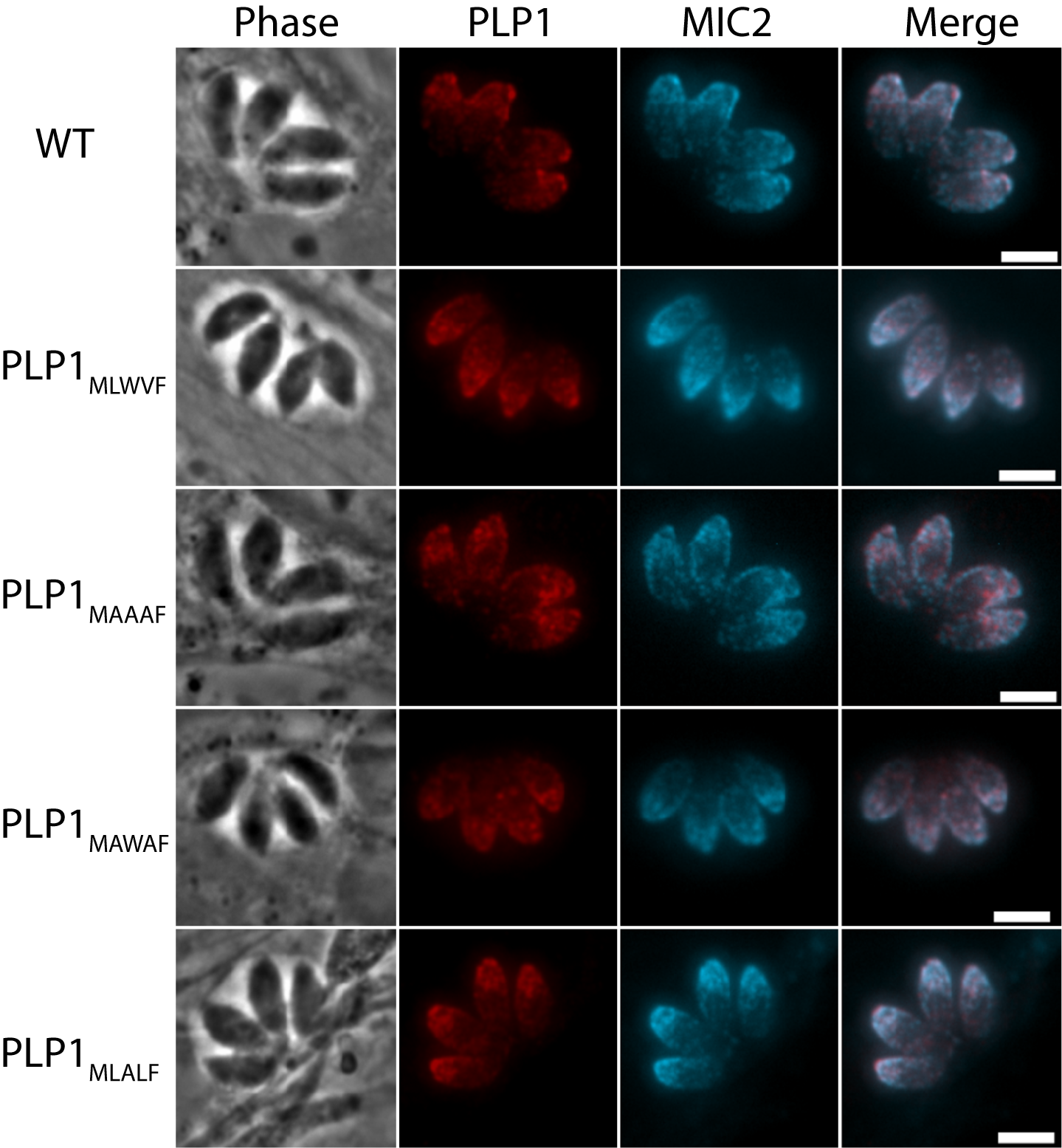

Supplement: S5 Fig — Host cells were inoculated with mechanically lysed parasites and incubated for 30 hours at 37°C. Immunofluorescence microscopy was performed by staining with mouse anti-TgMIC2 (cyan) and rabbit anti-TgPLP1 (red) antibodies and imaged via immunofluorescence microscopy. (TIF) [file ppat.1007476.s005.tif]
