# Supplementary material for: Structural basis of Toxoplasma gondii perforin-like protein 1 membrane interaction and activity during egress
Source: PLoS Pathog. 2018 Dec 4;14(12):e1007476. doi: 10.1371/journal.ppat.1007476 (PMC6294395; doi:10.1371/journal.ppat.1007476)
Supplement: S1 Table — (DOCX) [file ppat.1007476.s007.docx]

| Number | PDB-chain | Dali-Z | % ID | Molecule Description |
| --- | --- | --- | --- | --- |
| 1 | 5ouo-A | 48.5 | 100 | Perforin-like protein 1 |
| 2 | 5vlp-A | 6.0 | 17 | Proprotein convertase subtilisin/kexin type 9 |
| 3 | 5a3k-A | 3.9 | 10 | Putative pteridine-dependent dioxygenase |
