# Supplementary material for: Structural basis of Toxoplasma gondii perforin-like protein 1 membrane interaction and activity during egress
Source: PLoS Pathog. 2018 Dec 4;14(12):e1007476. doi: 10.1371/journal.ppat.1007476 (PMC6294395; doi:10.1371/journal.ppat.1007476)
Supplement: S1 Structure — (PDF) [file ppat.1007476.s008.pdf]

Full wwPDB X-ray Structure Validation Report (i)

May 8, 2018 – 02:56 PM EDT

PDB ID	:	6D7A
Title	:	Structure of T. gondii PLP1 beta-rich domain
Authors	:	Guerra, A.J.; Koropatkin, N.M.; Wawrzak, Z.; Bahr, C.M.E.; Carruthers, V.B.
Deposited on	:	2018-04-24
Resolution	:	1.13 Å(reported)

This is a Full wwPDB X-ray Structure Validation Report for a publicly released PDB entry.

We welcome your comments at *validation@mail.wwpdb.org*
A user guide is available at
https://www.wwpdb.org/validation/2017/XrayValidationReportHelp
with specific help available everywhere you see the (i) symbol.

The following versions of software and data (see references (i)) were used in the production of this report:

MolProbity	:	4.02b-467
Xtriage (Phenix)	:	1.13
EDS	:	rb-20031172
Percentile statistics	:	20171227.v01 (using entries in the PDB archive December 27th 2017)
Refmac	:	5.8.0158
CCP4	:	7.0 (Gargrove)
Ideal geometry (proteins)	:	Engh & Huber (2001)
Ideal geometry (DNA, RNA)	:	Parkinson et al. (1996)
Validation Pipeline (wwPDB-VP)	:	rb-20031172

1 Overall quality at a glance (i)

The following experimental techniques were used to determine the structure:
X-RAY DIFFRACTION

The reported resolution of this entry is 1.13 Å.

Percentile scores (ranging between 0-100) for global validation metrics of the entry are shown in the following graphic. The table shows the number of entries on which the scores are based.

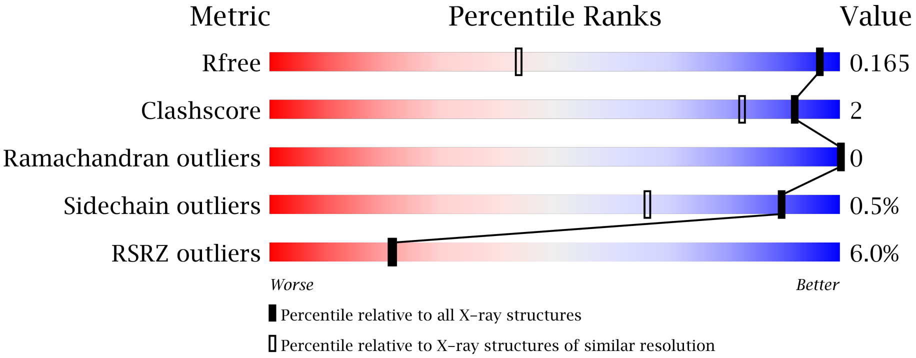

Metric	Whole archive (#Entries)	Similar resolution (#Entries, resolution range(Å))
R_{free}	111664	1189 (1.16-1.08)
Clashscore	122126	1004 (1.14-1.10)
Ramachandran outliers	120053	1188 (1.16-1.08)
Sidechain outliers	120020	1185 (1.16-1.08)
RSRZ outliers	108989	1165 (1.16-1.08)

The table below summarises the geometric issues observed across the polymeric chains and their fit to the electron density. The red, orange, yellow and green segments on the lower bar indicate the fraction of residues that contain outliers for >=3, 2, 1 and 0 types of geometric quality criteria. A grey segment represents the fraction of residues that are not modelled. The numeric value for each fraction is indicated below the corresponding segment, with a dot representing fractions <=5% The upper red bar (where present) indicates the fraction of residues that have poor fit to the electron density. The numeric value is given above the bar.

Mol	Chain	Length	Quality of chain
1	A	359	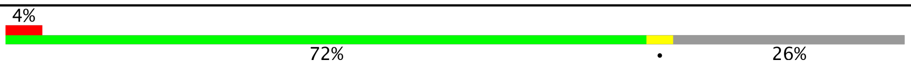
1	B	359	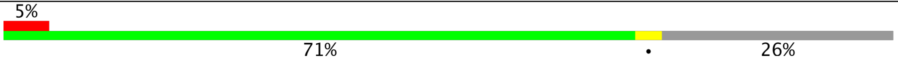

2 Entry composition

There are 3 unique types of molecules in this entry. The entry contains 8834 atoms, of which 4056 are hydrogens and 0 are deuteriums.

In the tables below, the ZeroOcc column contains the number of atoms modelled with zero occupancy, the AltConf column contains the number of residues with at least one atom in alternate conformation and the Trace column contains the number of residues modelled with at most 2 atoms.

- Molecule 1 is a protein called Perforin-like protein 1.

Mol	Chain	Residues	Atoms						ZeroOcc	AltConf	Trace
			Total	C	H	N	O	S			
1	A	266	4099	1292	2033	353	399	22	0	18	0
1	B	265	4077	1288	2023	351	394	21	0	14	0

There are 4 discrepancies between the modelled and reference sequences:

Chain	Residue	Modelled	Actual	Comment	Reference
A	805	SER	-	expression tag	UNP A1E348
A	806	ASN	-	expression tag	UNP A1E348
B	805	SER	-	expression tag	UNP A1E348
B	806	ASN	-	expression tag	UNP A1E348

- Molecule 2 is SODIUM ION (three-letter code: NA) (formula: Na).

Mol	Chain	Residues	Atoms		ZeroOcc	AltConf
			Total	Na		
2	B	2	2	2	0	0
2	A	2	2	2	0	0

- Molecule 3 is water.

Mol	Chain	Residues	Atoms		ZeroOcc	AltConf
			Total	O		
3	A	330	330	330	0	0
3	B	324	324	324	0	0

3 Residue-property plots (i)

These plots are drawn for all protein, RNA and DNA chains in the entry. The first graphic for a chain summarises the proportions of the various outlier classes displayed in the second graphic. The second graphic shows the sequence view annotated by issues in geometryand electron density. Residues are color-coded according to the number of geometric quality criteria for which they contain at least one outlier: green = 0, yellow = 1, orange = 2 and red = 3 or more. A red dot above a residue indicates a poor fit to the electron density (RSRZ > 2). Stretches of 2 or more consecutive residues without any outlier are shown as a green connector. Residues present in the sample, but not in the model, are shown in grey.

- Molecule 1: Perforin-like protein 1

Chain A:

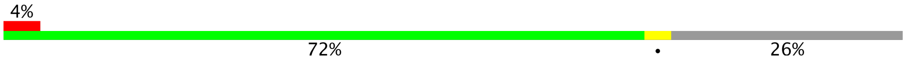

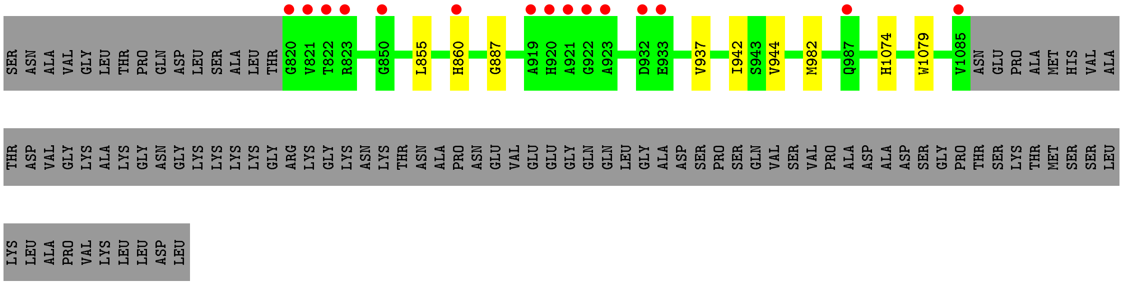

- Molecule 1: Perforin-like protein 1

Chain B:

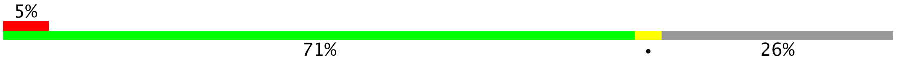

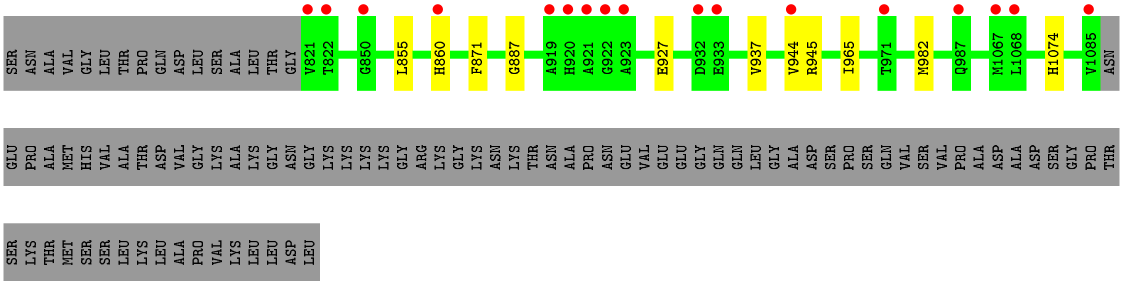

4 Data and refinement statistics (i)

Property	Value	Source
Space group	C 1 2 1	Depositor
Cell constants<br>a, b, c, α, β, γ	101.98Å 50.85Å 105.34Å<br>90.00° 90.13° 90.00°	Depositor
Resolution (Å)	24.88 – 1.13<br>24.88 – 1.13	Depositor<br>EDS
% Data completeness<br>(in resolution range)	94.9 (24.88-1.13)<br>94.9 (24.88-1.13)	Depositor<br>EDS
R_{merge}	0.08	Depositor
R_{sym}	(Not available)	Depositor
$< I/\sigma(I) >$ [1]	1.75 (at 1.13Å)	Xtriage
Refinement program	PHENIX	Depositor
R, R_{free}	0.141 , 0.164<br>0.143 , 0.165	Depositor<br>DCC
R_{free} test set	9508 reflections (4.97%)	wwPDB-VP
Wilson B-factor (Å^2)	10.1	Xtriage
Anisotropy	0.157	Xtriage
Bulk solvent k_{sol}(e/Å^3), B_{sol}(Å^2)	0.44 , 55.4	EDS
L-test for twinning[2]	$< \|L\| >$ = 0.51, $< L^2 >$ = 0.34	Xtriage
Estimated twinning fraction	0.016 for -h,-k,l	Xtriage
F_o,F_c correlation	0.98	EDS
Total number of atoms	8834	wwPDB-VP
Average B, all atoms (Å^2)	17.0	wwPDB-VP

Xtriage's analysis on translational NCS is as follows: *The analyses of the Patterson function reveals a significant off-origin peak that is 27.66 % of the origin peak, indicating pseudo-translational symmetry. The chance of finding a peak of this or larger height randomly in a structure without pseudo-translational symmetry is equal to 2.1148e-03. The detected translational NCS is most likely also responsible for the elevated intensity ratio.*

[1] Intensities estimated from amplitudes.

[2] Theoretical values of $< |L| >$, $< L^2 >$ for acentric reflections are 0.5, 0.333 respectively for untwinned datasets, and 0.375, 0.2 for perfectly twinned datasets.

5 Model quality (i)

5.1 Standard geometry (i)

Bond lengths and bond angles in the following residue types are not validated in this section: NA

The Z score for a bond length (or angle) is the number of standard deviations the observed value is removed from the expected value. A bond length (or angle) with $|Z| > 5$ is considered an outlier worth inspection. RMSZ is the root-mean-square of all Z scores of the bond lengths (or angles).

Mol	Chain	Bond lengths		Bond angles	
		RMSZ	#|Z| >5	RMSZ	#|Z| >5
1	A	0.39	0/2165	0.65	0/2930
1	B	0.41	0/2139	0.66	0/2895
All	All	0.40	0/4304	0.65	0/5825

There are no bond length outliers.

There are no bond angle outliers.

There are no chirality outliers.

There are no planarity outliers.

5.2 Too-close contacts (i)

In the following table, the Non-H and H(model) columns list the number of non-hydrogen atoms and hydrogen atoms in the chain respectively. The H(added) column lists the number of hydrogen atoms added and optimized by MolProbity. The Clashes column lists the number of clashes within the asymmetric unit, whereas Symm-Clashes lists symmetry related clashes.

Mol	Chain	Non-H	H(model)	H(added)	Clashes	Symm-Clashes
1	A	2066	2033	2023	6	0
1	B	2054	2023	2026	8	0
2	A	2	0	0	0	0
2	B	2	0	0	0	0
3	A	330	0	0	0	0
3	B	324	0	0	0	0
All	All	4778	4056	4049	14	0

The all-atom clashscore is defined as the number of clashes found per 1000 atoms (including hydrogen atoms). The all-atom clashscore for this structure is 2.

All (14) close contacts within the same asymmetric unit are listed below, sorted by their clash

magnitude.

Atom-1	Atom-2	Interatomic distance (Å)	Clash overlap (Å)
1:B:927[B]:GLU:CG	1:B:965:ILE:HD11	2.20	0.71
1:B:927[B]:GLU:HG3	1:B:965:ILE:HD11	1.78	0.66
1:B:944[A]:VAL:HG12	1:B:945:ARG:N	2.19	0.57
1:B:855:LEU:HD22	1:B:982[A]:MET:SD	2.48	0.54
1:A:982[B]:MET:HE1	1:A:1079:TRP:HH2	1.74	0.51
1:A:937:VAL:HB	1:A:982[B]:MET:HG3	1.95	0.48
1:A:855:LEU:HD22	1:A:982[A]:MET:SD	2.54	0.48
1:B:860[B]:HIS:CD2	1:B:887:GLY:HA3	2.50	0.47
1:B:871:PHE:CE1	1:B:944[A]:VAL:HG11	2.50	0.46
1:A:860[B]:HIS:CD2	1:A:887:GLY:HA3	2.52	0.45
1:B:937:VAL:HB	1:B:982[B]:MET:HG3	1.99	0.44
1:B:871:PHE:CZ	1:B:944[A]:VAL:HG11	2.54	0.42
1:A:942:ILE:HG22	1:A:944:VAL:HG13	2.01	0.42
1:A:855:LEU:HD22	1:A:982[B]:MET:SD	2.60	0.41

There are no symmetry-related clashes.

5.3 Torsion angles (i)

5.3.1 Protein backbone (i)

In the following table, the Percentiles column shows the percent Ramachandran outliers of the chain as a percentile score with respect to all X-ray entries followed by that with respect to entries of similar resolution.

The Analysed column shows the number of residues for which the backbone conformation was analysed, and the total number of residues.

Mol	Chain	Analysed	Favoured	Allowed	Outliers	Percentiles	
1	A	282/359 (79%)	271 (96%)	11 (4%)	0	100	100
1	B	277/359 (77%)	271 (98%)	6 (2%)	0	100	100
All	All	559/718 (78%)	542 (97%)	17 (3%)	0	100	100

There are no Ramachandran outliers to report.

5.3.2 Protein sidechains (i)

In the following table, the Percentiles column shows the percent sidechain outliers of the chain as a percentile score with respect to all X-ray entries followed by that with respect to entries of similar

resolution.

The Analysed column shows the number of residues for which the sidechain conformation was analysed, and the total number of residues.

Mol	Chain	Analysed	Rotameric	Outliers	Percentiles	
1	A	236/292 (81%)	235 (100%)	1 (0%)	92	76
1	B	232/292 (80%)	231 (100%)	1 (0%)	92	76
All	All	468/584 (80%)	466 (100%)	2 (0%)	90	76

All (2) residues with a non-rotameric sidechain are listed below:

Mol	Chain	Res	Type
1	A	1074	HIS
1	B	1074	HIS

Some sidechains can be flipped to improve hydrogen bonding and reduce clashes. There are no such sidechains identified.

5.3.3 RNA (i)

There are no RNA molecules in this entry.

5.4 Non-standard residues in protein, DNA, RNA chains (i)

There are no non-standard protein/DNA/RNA residues in this entry.

5.5 Carbohydrates (i)

There are no carbohydrates in this entry.

5.6 Ligand geometry (i)

Of 4 ligands modelled in this entry, 4 are monoatomic - leaving 0 for Mogul analysis.

There are no bond length outliers.

There are no bond angle outliers.

There are no chirality outliers.

There are no torsion outliers.

There are no ring outliers.

No monomer is involved in short contacts.

5.7 Other polymers (i)

There are no such residues in this entry.

5.8 Polymer linkage issues (i)

There are no chain breaks in this entry.

6 Fit of model and data

6.1 Protein, DNA and RNA chains

In the following table, the column labelled '#RSRZ> 2' contains the number (and percentage) of RSRZ outliers, followed by percent RSRZ outliers for the chain as percentile scores relative to all X-ray entries and entries of similar resolution. The OWAB column contains the minimum, median, 95th percentile and maximum values of the occupancy-weighted average B-factor per residue. The column labelled 'Q< 0.9' lists the number of (and percentage) of residues with an average occupancy less than 0.9.

Mol	Chain	Analysed	<RSRZ>	#RSRZ>2			OWAB(Å^2)	Q<0.9
1	A	266/359 (74%)	-0.12	15 (5%)	24	23	7, 12, 29, 48	0
1	B	265/359 (73%)	-0.05	17 (6%)	19	19	7, 11, 30, 46	0
All	All	531/718 (73%)	-0.08	32 (6%)	22	21	7, 12, 30, 48	0

All (32) RSRZ outliers are listed below:

Mol	Chain	Res	Type	RSRZ
1	B	821	VAL	9.5
1	B	822	THR	8.8
1	B	944[A]	VAL	7.6
1	A	921	ALA	6.9
1	B	1085	VAL	6.4
1	B	860[A]	HIS	6.4
1	A	920	HIS	6.2
1	A	860[A]	HIS	6.0
1	A	919	ALA	5.8
1	B	920	HIS	5.5
1	B	921	ALA	5.3
1	A	932	ASP	4.7
1	B	1068	LEU	4.3
1	B	923	ALA	4.1
1	A	1085	VAL	4.0
1	B	1067	MET	3.8
1	B	932	ASP	3.7
1	A	820	GLY	3.6
1	B	919	ALA	3.6
1	A	923	ALA	3.5
1	A	821	VAL	3.4
1	A	987	GLN	3.0
1	A	933	GLU	3.0
1	B	933	GLU	2.8

Continued on next page...

Continued from previous page...

Mol	Chain	Res	Type	RSRZ
1	B	850	GLY	2.6
1	B	922	GLY	2.5
1	B	971	THR	2.4
1	A	922	GLY	2.3
1	A	850	GLY	2.2
1	A	823	ARG	2.2
1	B	987	GLN	2.2
1	A	822	THR	2.1

6.2 Non-standard residues in protein, DNA, RNA chains ⓘ

There are no non-standard protein/DNA/RNA residues in this entry.

6.3 Carbohydrates ⓘ

There are no carbohydrates in this entry.

6.4 Ligands ⓘ

In the following table, the Atoms column lists the number of modelled atoms in the group and the number defined in the chemical component dictionary. The B-factors column lists the minimum, median, 95th percentile and maximum values of B factors of atoms in the group. The column labelled 'Q< 0.9' lists the number of atoms with occupancy less than 0.9.

Mol	Type	Chain	Res	Atoms	RSCC	RSR	B-factors($Å^2$)	Q<0.9
2	NA	A	1202	1/1	1.00	0.04	9,9,9,9	0
2	NA	B	1202	1/1	1.00	0.05	9,9,9,9	0
2	NA	B	1201	1/1	1.00	0.03	14,14,14,14	0
2	NA	A	1201	1/1	1.00	0.04	15,15,15,15	0

6.5 Other polymers ⓘ

There are no such residues in this entry.
